# Assessing standards for prevention of early onset group B streptococcal (GBS) disease in Ireland

**DOI:** 10.1007/s11845-021-02639-7

**Published:** 2021-05-14

**Authors:** Alex Dakin, Wendy Ferguson, Richard Drew, Naomi McCallion, Mary F. Higgins, Maeve Eogan

**Affiliations:** 1 Obstetrics and Gynaecology, Rotunda Hospital, Dublin, Ireland; 2 Paediatrics, Rotunda Hospital, Dublin, Ireland; 3 Microbiology, Dublin, Ireland; 4 Obstetrics and Gynaecology, National Maternity Hospital, Dublin, Ireland

**Keywords:** Group B streptococcus, Intrapartum antibiotic prophylaxis, Polymerase chain reaction, Screening

## Abstract

**Background:**

Early onset group B streptococcal (GBS) disease can cause significant neonatal morbidity and mortality. There is currently no Irish national guideline for GBS screening, and protocols vary across maternity units. Polymerase chain reaction (PCR) testing at induction or labour onset informs triage for antibiotic prophylaxis; however, there are human and infrastructural resource requirements to enable widespread implementation.

**Aim:**

Our aim was to identify current standard practices for GBS prevention in Irish obstetric and neonatal services and to utilise this data to inform the need for, and potential impact of implementation of, a national guideline.

**Methods:**

A questionnaire on GBS screening, management and existing resources was completed by an informed staff member from each of the 19 Irish maternity units, including questions regarding timing and method of screening, antibiotic usage, and neonatal management.

**Results:**

One unit (5.2%) performs routine GBS screening at 35–37 weeks of gestation. Twelve units (63%) screen for GBS following spontaneous rupture of membranes (SROM) after 37 weeks, of which two (17%) perform PCR and ten (83%) culture testing. Seventeen units (89.3%) have access to a GeneXpert PCR machine, and of these, two (11.7%) use the machine for rapid GBS testing. Two units screen patients for GBS at either the start of labour or induction of labour. Four units (21%) use the neonatal early onset sepsis (EOS) calculator. Sixteen units (84%) do not treat asymptomatic infants born to GBS-positive mothers.

**Conclusion:**

There is a lack of consistency in the methods for GBS screening and disease prevention across the country, highlighting the need for a national guideline accompanied by an implementation plan and budget to standardise care.

## Introduction

*Streptococcus agalactiae*, or group B streptococcus (GBS), is a facultative gram-positive organism and a commensal organism of the gastrointestinal and genital tracts. Ten to thirty percent of pregnant women are colonised with GBS [1] [2], and it can be transmitted to the neonate during delivery and can cause neonatal infection. Maternal complications of GBS include sepsis, urinary tract infection, intra-amniotic infection, endometritis and preterm labour. Fetal complications include stillbirth and neonatal invasive disease, including meningitis, sepsis, pneumonia and death [3]. Early onset GBS (EOGBS) disease, occurring from birth to day 6 of life, progresses rapidly, presenting with sepsis in 63% or pneumonia in 26%, whereas late onset GBS (LOGBS) disease occurs from days 7 to 90 of life, and can have a more indolent onset, causing meningitis in 43% or other focal infection in another 7% [4]. GBS is the leading cause of bacterial meningitis in infants in the UK [5].

Several risk factors are known to increase transmission of EOGBS disease. Risk factors such as maternal pyrexia, prolonged rupture of membranes for greater than 18 h and preterm delivery were present in 20.4% of women in the UK [1]. A study of the burden of GBS disease in the UK and Ireland found that 58% of cases of EOGBS, and 73% of the deaths from EOGBS, had one or more of the following risk factors — preterm delivery, prolonged rupture of membranes for longer than 18 h and antenatal genitourinary carriage of GBS. Over one-third (37%) of cases were associated with preterm delivery before 37 weeks, less than half (44%) had prolonged rupture of membranes for longer than 18 h and only a small number (4%) had known genital carriage of GBS during pregnancy [4]. However, presence of risk factors is not entirely predictive of carriage of GBS — a systematic review found that only 28.9% of women with clinical risk factors actually carried GBS and that 19% of women with no clinical risk factors carried GBS [6].

Of babies born to GBS colonised mothers, 36% become colonised with GBS at birth, and 3% of colonised babies develop EOGBS bacteraemia [6]. A national surveillance study carried out by the Royal College of Obstetricians and Gynaecologists (RCOG) states that the incidence of sepsis due to GBS is 1.3 in 1000 live births in the UK [7]. Invasive GBS disease carries a 10% infant mortality rate [8]. EOGBS disease has a higher mortality rate than LOGBS disease (10.5% vs 8%) [4]. The mortality rate is significantly higher in infants born prematurely — 15.2% in neonates born before 33 weeks, compared to 6.4% in neonates born after 37 weeks [4]. The incidence of culture-confirmed EOGBS appears to be rising in the UK from 0.48 per 1000 live births in 2000 to 0.57 per 1000 live births in 2014 [5]; this is despite a clinical risk-factor-based intrapartum antibiotic (IAP) protocol introduced in 2003 [9].

IAP reduces the rates of EOGBS disease [10]. Multiple different methods are employed in different countries to determine who requires IAP to reduce the rate of EOGBS disease. A review of GBS policies worldwide showed that the majority (63%) of countries surveyed had an IAP policy, over half (58%) used microbiological screening, and many (42%) used a clinical risk-factor-based policy [11]. In the USA, the Center for Disease Control (CDC), American College of Obstetricians and Gynaecologists (ACOG) and American Society for Microbiology (ASM) recommend universal antenatal screening, comprising culture testing for GBS at 36–38 weeks and providing IAP for those who are positive, in addition to the use of IAP because of GBS bacteriuria in the current pregnancy or a history of previous GBS-infected neonate [8, 12, 13]. This approach is also recommended by the Royal Australian and New Zealand College of Obstetricians and Gynaecologists (RANZCOG) [14] and has been implemented by several European countries, including France [15]. In contrast, the RCOG do not recommend universal screening. They recommend IAP for women at increased risk, for example, a previous baby with GBS disease, GBS cultured on high vaginal swab (HVS) or mid-stream urine (MSU) in the current pregnancy, prolonged rupture of membranes longer than 18 h, maternal pyrexia and preterm labour [9]. Ireland does not have a national guideline, and clinicians here generally follow the RCOG advice. Several studies support the use of universal screening policies, stating that they lead to a lower incidence of EOGBS sepsis compared to risk-based policies [16] [17]; however, there is no randomised controlled trial evidence to support this strategy, which may be why the UK National Screening Committee and RCOG recommend the risk-factor-based approach. The low incidence of EOGBS neonatal sepsis in the UK without universal screening [18] lends support to this decision.

The potential concern with antenatal culture screening in the third trimester is the transient nature of GBS colonisation. A systematic review of the timing of GBS screening stated that 30% of women with a positive GBS culture at 35 weeks or later had changed to a negative status by birth [19]. This could result in either under-treatment or over-treatment with IAP [20]. Another potential problem with the antenatal screening approach is preterm delivery before routine GBS culture testing. Real-time polymerase chain reaction (PCR) testing for GBS at the onset of labour performed in either the laboratory or the delivery suite is a potential solution to these problems. There are several GBS PCR systems available, such as Roche LightCycler and cobas Liat, and the Cepheid Xpert GBS system is already in use Ireland. The Cepheid Xpert GBS PCR test has a sensitivity of 89–99%, and a specificity of 90–99% compared to antenatal culture [21–25], and PCR testing has led to half of the women with an antepartum-positive culture avoiding unnecessary antibiotic usage [21]. A previous study by our group showed that rapid PCR testing had a sensitivity of 93.1% and specificity of 96.67%, compared to routine culture, and there was a similar rate of GBS carriage (18.98% by culture and 19.62% by PCR testing) [26]. Of women who received IAP for prolonged rupture of membranes based on the risk-factor-based approach, only 31.6% were GBS carriers by PCR testing. Conversely, only 19.4% of the GBS-positive women who needed IAP qualified for it based on their individual risk factors [26]. The European Consensus Conference Group on intrapartum GBS screening and antibiotic prophylaxis recommend intrapartum PCR testing of all women to determine provision of IAP, with the exception of women with a previous child with invasive GBS disease or who have GBS bacteriuria in the current pregnancy, who should receive IAP regardless [20]. In the case of a negative PCR result, their recommendation is that IAP should be only be given with prolonged rupture of membranes longer than 18 h, or in the presence of maternal pyrexia [20].

The aim of this study is to identify current standards of GBS screening and prevention across the 19 Irish maternity units, identify differences and similarities between units, and investigate the need for a national guideline and implementation programme.

## Methods

This is a cross-sectional study evaluating the protocols for screening and management of GBS in the maternity units across the country. A questionnaire was formulated comprising questions regarding timing and method of GBS screening, antibiotic usage, neonatal management and existing resources. These were completed by an informed staff member at each of the 19 maternity units in September 2020. This data was then collated into an Excel spreadsheet; results were analysed, and protocols compared against each other.

## Results

Table 1 outlines the nineteen maternity units, subdivided into large (greater than 6000 births per year), medium (2001–6000 births per year) and small units (less than 2000 births per year) [27]. As per the Central Statistics Office Birth Register there were 61,538 births in 2017, 53% of these occurred in large units, 23% occurred in medium-sized units, and the remainder occurred in small units. [28].

**Table 1:** Irish maternity hospitals subdivided by yearly birth rate (as per NPEC report 2017) [27]

Births/year	Hospital	Births/year	Hospital
> 6001	Rotunda Hospital	< 2000	Sligo University Hospital
	National Maternity Hospital		Mayo University Hospital
	Coombe Women and Infants Hospital		Portiuncula Hospital
	Cork University Maternity Hospital		Letterkenny University Hospital
2001–6000	University Maternity Hospital, Limerick		Cavan General Hospital
	Our Lady of Lourdes Hospital, Drogheda		Midland Regional Hospital Portlaoise
	Galway University Hospital		St Luke’s Hospital, Kilkenny
	University Hospital Waterford		Wexford General Hospital
	Midland Regional Hospital Mullingar		South Tipperary General Hospital
			University Hospital Kerry

### Timing and method of GBS screening

One unit (5.2%) carries out routine antenatal screening for GBS between 35 and 37 weeks of gestation, in line with CDC guidelines [8]. All nineteen maternity units carry out routine GBS screening in cases of preterm pre-labour rupture of membranes in line with Royal College of Physicians of Ireland guidelines [29].

Fewer than half (47%) of units (9/19) undertake screening for GBS in all cases of spontaneous rupture of membranes (SROM) at term (i.e. greater than 37 weeks). Of these, two are larger units, and ten are smaller units. A further three units (16%) perform GBS screening only in cases where sterile speculum examination is required for the diagnosis of SROM — one medium-sized unit and two smaller units. The remaining seven units (37%) do not perform screening for GBS in cases of SROM at term. This is further broken down into two larger units, four medium units and one smaller unit.

Of the 12 units that carry out GBS screening in cases of SROM at term, two (17%) perform rapid polymerase chain reaction (PCR) testing — these are two of the larger units. Ten units (83%) perform culture testing, including nine smaller units and one medium unit. The two units that have rapid GBS screening available give intrapartum antibiotic prophylaxis (IAP) and augment labour with oxytocin if GBS-positive, and they do not give IAP if GBS-negative, regardless of duration of ruptured membranes.

**Fig. 1:**
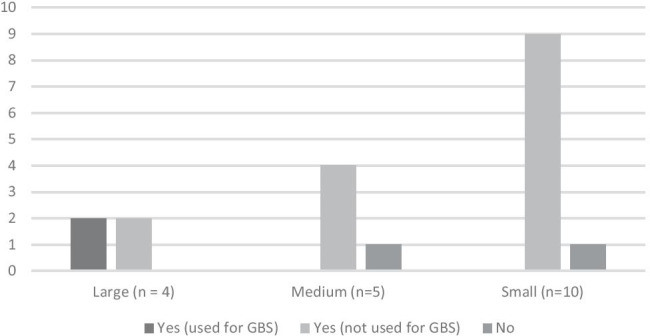
Availability of GeneXpert machine, subdivided by size of maternity unit (small < 2000 births/year, medium 2001–6000 births/year, large > 6000 births/year)

The majority (17/19 units (89.3%)) have a GeneXpert PCR machine in their hospital. Of these, two (11.7%) use this machine for rapid GBS testing. Figure 1 outlines the GeneXpert machine availability stratified by size of maternity unit.

### Antibiotic use

All nineteen maternity units give intravenous benzylpenicillin as IAP in patients with no penicillin allergy. Seven of nineteen units (37%) use cephalosporins for mild penicillin allergy, and the remainder of units do not differentiate between types of penicillin allergy in determining choice of antibiotic. In the case of severe penicillin allergy 16 units (85%) give clindamycin, one (5%) gives vancomycin, one (5%) gives ceftriaxone and one (5%) gives teicoplanin.

### Neonatal management

**Fig. 2:**
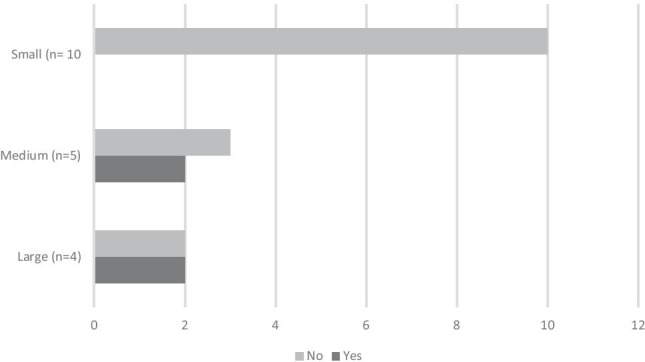
Use of early onset sepsis calculator subdivided by size of maternity unit (small < 2000 births/year, medium 2001–6000 births/year, large > 6000 births/year)

As Fig. 2 demonstrates, four of the nineteen units (21%) use the Kaiser-Permanente early onset sepsis (EOS) calculator to determine need for antibiotic treatment in GBS-exposed infants.

**Fig. 3:**
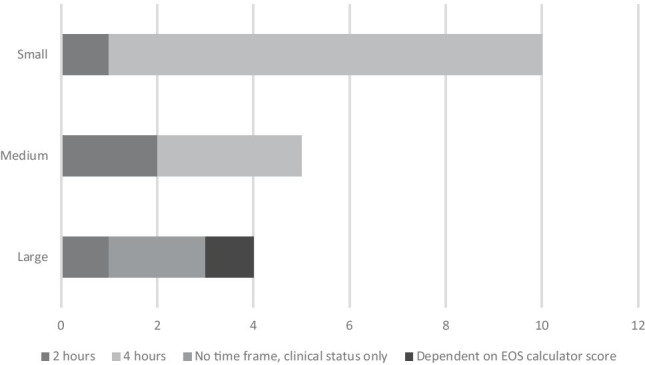
Duration of maternal intrapartum antibiotic prophylaxis use pre-delivery to provide adequate neonatal cover, subdivided by size of maternity unit (small < 2000 births/year, medium 2001–6000 births/year, large > 6000 births/year)

More than half (63%) of units (12/19) determine 4 h of maternal antibiotic use prior to delivery as “adequate prophylactic cover” for neonatal GBS. Four units (21%) units define 2 h as “adequate cover”. Two units (11%) do not specify a timeframe, and decision for antibiotic use in the neonate is determined by clinical status only. One unit (5%) makes their decision for antibiotic use in the neonate based on the EOS calculator score. This is further stratified by size of maternity unit in Fig. 3.

**Fig. 4:**
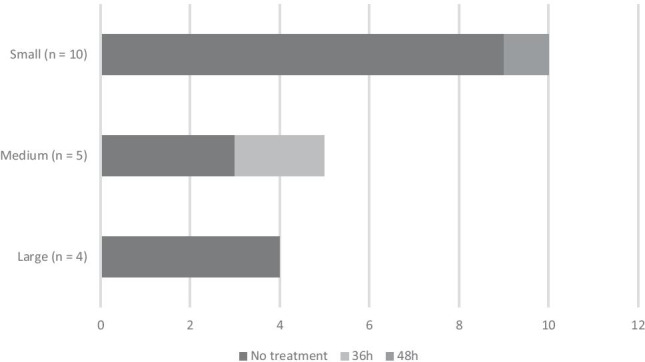
Antibiotic duration for asymptomatic infant born to mother colonised with group B streptococcus, subdivided by size of maternity unit (small < 2000 births/year, medium 2001–6000 births/year, large > 6000 births/year)

Figure 4 illustrates intravenous antibiotic duration for infants who are asymptomatic, with no risk factors, but born to GBS-positive women, who had been adequately covered with IAP. Eighty-four percent of units (16/19) do not give prophylactic antibiotics, 11% (2/19) will give antibiotics for 36 h, and the remaining unit (1/19, 5%) will give antibiotics for 48 h.

## Discussion

As yet, there are no prospective randomised controlled trials comparing the universal screening method with the risk-factor-based approach [10]. In general, Ireland follows the risk-factor-based approach recommended by the RCOG [9], with the exception of one unit which carries out universal antenatal screening at 35–37 weeks.

There is significant variation in practice between the different maternity units, in terms of GBS screening approach, testing, antibiotic usage and neonatal management. In our current system, trainees rotate through different hospitals on a yearly basis. It is undesirable to have significant variability in management between units, and if a national guideline was implemented, this would ensure that management was standardised across units and greatly reduces the possibility for management errors. This would improve the standard of care for our patients.

This study shows variation between units regarding antibiotic choice in the case of penicillin allergy. RCOG recommend cephalosporin use in mild penicillin allergy, and vancomycin in severe penicillin allergy [9]. ACOG advice differs slightly from this in the management of severe penicillin allergy; they recommend clindamycin, but only if the GBS isolate is known to be susceptible to clindamycin; otherwise, vancomycin is recommended [13].

Some units (21%) use the Kaiser-Permanente EOS calculator in order to determine the need for antibiotic treatment amongst neonates exposed to GBS. Several studies show that this reduces empirical antibiotic usage, without a resultant increase in EOGBS disease [30, 31]. Overuse of antibiotics leads to antibiotic resistance, and this is a growing problem worldwide, in both maternal and neonatal cohorts [32, 33]. Providing antibiotics to those who test positive for GBS intrapartum will lead to reduced levels of unnecessary antibiotic prophylaxis, as opposed to the risk-factor-based approach.

Intrapartum PCR testing for GBS is employed in two hospitals in Ireland, in line with recommendations from the European Consensus Group [20]. Ten units screen for GBS in the case of spontaneous rupture of membranes at term with routine culture testing from high vaginal swab; however, the result would not be available in time to impact intrapartum or early neonatal management.

A barrier to intrapartum PCR testing is the significant resource implications. A GeneXpert GBS cartridge costs £38.80, and the diagnostic system ranges in cost from £17,602 for a single-module system, to £118,119 for a 16-module system, as per a review by NICE [34]. A diagnostic accuracy study in 2009 on the Cepheid GeneXpert system versus bacterial culture testing and found that PCR was not cost-effective based on the sensitivity, specificity and cost at the time [2]. A more recent study showed that PCR significantly reduces the percentage of unnecessarily treated women compared to culture (4.5 and 13.6%, respectively) [35]. A comparison of EOGBS rates in a French hospital prior to and after introduction of an intrapartum PCR screening protocol demonstrated that antenatal culture had a positive predictive value of 58.3% compared with intrapartum PCR screening. After the introduction of intrapartum PCR testing, the rate of proven EOGBS disease cases decreased from 1.01/1000 live births to 0.21/1000 live births, and the rates of probable EOGBS cases from 2.8/1000 to 0.73/1000. Intrapartum PCR testing also reduced the number of days of antibiotic usage for EOGBS by 60%. The authors suggested that the cost of the GeneXpert machine would be fully balanced by the EOGBS disease avoided [36].

Intrapartum PCR test implementation in Ireland would involve significant costs of setting up machines in all units, GBS-specific cartridges, training staff, and ensuring sufficient laboratory staffing levels to provide PCR testing 24/7. In addition to the two maternity units in Ireland that carry out GBS PCR testing, a further fifteen units have access to a GeneXpert machine which is utilised for PCR testing for other viruses, for example, SARS CoV2 and influenza viruses. The additional cost of enabling these machines for GBS PCR testing and the resultant staff training and rostering could be balanced by the reduction in rates of actual or possible EOGBS disease, and the resultant cost saving in neonatal care [36]. Introduction of intrapartum PCR testing will result in an increased workload for laboratory staff. One option to deal with the increase in number of tests performed is to train midwifery staff to use a point-of-care machine on the labour ward [25]; however, this is not in current practice in Ireland and still requires medical scientist support (for example, to troubleshoot errors). An Irish study in a unit that has intrapartum PCR screening showed that 70% of women who would have been eligible for IAP due to prolonged rupture of membranes avoided antibiotic therapy following a negative PCR test; however, their test was not available out of hours [37]. In order for PCR testing to have its full impact on 24/7 maternity services, a result would have to be available around the clock. Overall, we do believe that there is a significant additional cost associated with the introduction of GBS PCR screening nationwide, but this is a worthwhile cost as it will lead to both a reduction in unnecessary antibiotic usage in both mothers and infants, and also a reduction in the rate of EOGBS disease, therefore partly offsetting the cost. [36].

This review of GBS protocols across the nineteen Irish maternity units has highlighted a wide variation in standards for GBS prevention nationally, demonstrated by differing methods used for GBS screening, type of IAP usage, and neonatal management. This highlights the need for development of a national GBS prevention guideline with a funded implementation strategy and auditable standards, in order to deliver high-quality preventative care across all maternity units in Ireland and to reduce the potentially devastating maternal, and particularly neonatal, effects of invasive GBS disease.
